# Wide Variation in the Use of Radiotherapy in the Management of Surgically Treated Rectal Cancer Across the English National Health Service

**DOI:** 10.1016/j.clon.2016.02.002

**Published:** 2016-08

**Authors:** E.J.A. Morris, P.J. Finan, K. Spencer, I. Geh, A. Crellin, P. Quirke, J.D. Thomas, S. Lawton, R. Adams, D. Sebag-Montefiore

**Affiliations:** ∗ Cancer Epidemiology Group, Leeds Institute of Cancer & Pathology, University of Leeds, St James's University Hospital, Leeds, UK; † John Goligher Colorectal Unit, St James's University Hospital, Leeds, UK; ‡ National Cancer Intelligence Network, London, UK; § Non-Surgical Oncology, Leeds Teaching Hospitals NHS Trust, St James's University Hospital, Leeds, UK; ¶ University Hospitals Birmingham NHS Foundation Trust, Queen Elizabeth Medical Centre, Birmingham, UK; ‖ Section of Pathology and Tumour Biology, Leeds Institute of Cancer & Pathology, University of Leeds, St James's University Hospital, Leeds, UK; ∗∗ National Cancer Registration Service, Northern and Yorkshire Office, St James's Institute of Oncology, St James's University Hospital, Leeds, UK; †† Knowledge and Intelligence Team (Northern and Yorkshire), St James's Institute of Oncology, St James's University Hospital, Leeds, UK; ‡‡ Cardiff University School of Medicine, Velindre Hospital, Cardiff, UK; §§ Section of Clinical Oncology, Leeds Institute of Cancer & Pathology, University of Leeds, St James's University Hospital, Leeds, UK

**Keywords:** Radiotherapy, rectal cancer, surgery

## Abstract

**Aims:**

Radiotherapy is an important treatment modality in the multidisciplinary management of rectal cancer. It is delivered both in the neoadjuvant setting and postoperatively, but, although it reduces local recurrence, it does not influence overall survival and increases the risk of long-term complications. This has led to a variety of international practice patterns. These variations can have a significant effect on commissioning, but also future clinical research. This study explores its use within the large English National Health Service (NHS).

**Materials and methods:**

Information on all individuals diagnosed with a surgically treated rectal cancer between April 2009 and December 2010 were extracted from the Radiotherapy Dataset linked to the National Cancer Data Repository. Individuals were grouped into those receiving no radiotherapy, short-course radiotherapy with immediate surgery (SCRT-I), short-course radiotherapy with delayed surgery (SCRT-D), long-course chemoradiotherapy (LCCRT), other radiotherapy (ORT) and postoperative radiotherapy (PORT). Patterns of use were then investigated.

**Results:**

The study consisted of 9201 individuals; 4585 (49.3%) received some form of radiotherapy. SCRT-I was used in 12.1%, SCRT-D in 1.2%, LCCRT in 29.5%, ORT in 4.7% and PORT in 2.3%. Radiotherapy was used more commonly in men and in those receiving an abdominoperineal excision and less commonly in the elderly and those with comorbidity. Significant and substantial variations were also seen in its use across all the multidisciplinary teams managing this disease.

**Conclusion:**

Despite the same evidence base, wide variation exists in both the use of and type of radiotherapy delivered in the management of rectal cancer across the English NHS. Prospective population-based collection of local recurrence and patient-reported early and late toxicity information is required to further improve patient selection for preoperative radiotherapy.

## Highlights

• Wide variation exists in both the use of and type of radiotherapy delivered in the management of rectal cancer.
• Population-based capture of local recurrence and toxicity information are required to further improve patient selection.

## Introduction

Radiotherapy is an established treatment modality in the multidisciplinary management of rectal cancer. In the 1990s, phase III trials reported reduced local recurrence and improved overall survival using a combination of postoperative chemotherapy and concurrent chemoradiation (CRT). Subsequently, randomised trials, mainly in Europe, showed a reduction in local recurrence with the preoperative addition of either a 1 week short course of radiotherapy or the addition of concurrent chemotherapy to a 5 week course of preoperative radiotherapy [1].

Two recently reported phase III trials have confirmed a halving of the rate of local recurrence when a 1 week short course of radiotherapy was added to surgical resection [2], [3]. Two phase III trials reported reduced local recurrence when preoperative CRT was compared with long-course radiotherapy alone [4], [5]. Local recurrence, acute and late toxicity were reduced when preoperative CRT was compared with postoperative CRT [6,^7^]. The combined results led to a major shift towards the use of preoperative radiotherapy in the form of short-course and CRT schedules. In parallel, improved surgical technique using total mesorectal excision led to low reported rates of local recurrence with surgery alone [8]. This finding was confirmed in the Medical Research Council CR07 trial, where the best planes of surgical excision resulted in the lowest rates of local recurrence [9].

Although preoperative radiotherapy can reduce the risk of local recurrence, it can also increase the risk of long-term side-effects when added to surgical resection [10], [11], [12], [13]. These long-term side-effects seem to be similar whether preoperative short-course radiotherapy or CRT is used [14], [15].

In the National Health Service (NHS) of England, weekly multidisciplinary team (MDT) meetings take place to review the clinical and radiological staging of all rectal cancer patients. Pelvic magnetic resonance imaging (MRI) is routinely used to determine the use of preoperative and postoperative radiotherapy in management, further increasing the complexity of decision making. International evidence suggests there is significant variation in the use of radiotherapy in the management of rectal cancer [16], [17], [18], [19], [20], but, unlike the UK, many countries do not routinely use MRI for pelvic staging, which may explain in part the variation observed. Little is known about the patterns of radiotherapy use in England.

This study explored the use of radiotherapy in surgically treated rectal cancer at a population level using the first available data from the national Radiotherapy Dataset (RTDS) [21]. These data are extracted and collated from all NHS linear accelerators. When combined with the information in the National Cancer Data Repository (NCDR) [22] these data enable patterns of management to be investigated [23], [24], [25] across the English NHS.

## Materials and Methods

All individuals diagnosed with a first primary rectal cancer between 1 April 2009 and 31 December 2010 and who underwent a major resection for the disease within the English NHS were identified (using standard algorithms) [24], [26] within the linked cancer registry and Hospital Episode Statistics (HES) component of the NCDR [22]. Information on age, gender, Dukes' stage of disease at diagnosis, tumour site, socioeconomic status (based on Index of Multiple Deprivation (IMD) income quintile) and survival were taken from the cancer registry component of the resource, whereas information on the type of surgery and hospital MDT of surgical management was taken from the HES component. A Charlson comorbidity score [27] was also derived for each individual based on the diagnostic reasons (excluding cancer) for any hospital admissions recorded in HES in the year before diagnosis (excluding any admission spanning the date of diagnosis). The cancer component of the score was then derived from the cancer registry information and combined with the hospital admission scores. Higher scores indicate greater comorbid disease and patients were grouped into Charlson score categories of 0, 1, 2 and ≥3.

To investigate patterns of use of radiotherapy, any records for this cohort of individuals within the RTDS (now also available in the NCDR) were identified. The RTDS contains information on every episode of radiotherapy delivered, but the dataset does not consistently capture whether the intent of the dose delivered was adjuvant, radical or palliative. In addition, the disease coding within the resources varies between centres and total attendances are captured rather than intended fractionation patterns. An individual may also have multiple summary RTDS records that overlap the same time period and seem to relate to the same episode of radiotherapy being delivered. An algorithm was therefore developed to identify neoadjuvant or adjuvant treatment records from the resource among all other episodes of radiotherapy administered to this rectal cancer population. First, only episodes of radiotherapy that the RTDS stated had been used to treat colorectal (ICD10 [28] C18-20), anal (C21) or an unspecified digestive cancer (C78, C80, D01 and D37) and occurred within a year of the date of surgery for each individual in the cohort were deemed eligible. If individuals had multiple episodes of radiotherapy delivered in overlapping time periods then the episode that recorded the highest number of attendances was retained, but the individual was flagged so that these multiple episodes were acknowledged. Individuals were then allocated to one of five groups based on the standard rectal radiotherapy regimens used in England and the total number of attendances they made to a radiotherapy centre. Those for whom there was no link to the RTDS were deemed to have received no neoadjuvant or adjuvant radiotherapy. Those who had attended a radiotherapy centre five times before surgery and for whom the time between the start of radiotherapy and surgery was 35 days or less were allocated to a short-course radiotherapy and immediate surgery category (SCRT-I). Those meeting the same attendance criteria, but where the interval between radiotherapy and surgery was greater than 35 days, were allocated to the short-course radiotherapy and delayed surgery category (SCRT-D). Those who attended for radiotherapy 25, 28 or 30 times were deemed to have undergone long-course chemoradiotherapy (LCCRT). In addition, those who had multiple radiotherapy records in the RTDS spanning the same time period where the maximum attendance in one of those records was 10 or more (but the addition of attendances in the other relevant records would exceed 25 attendances) were also allocated to LCCRT. Individuals who had attended for radiotherapy at a frequency different to these standard rectal fraction patterns were allocated to an ‘other’ radiotherapy category (ORT). Finally, individuals who received radiotherapy up to a year after their surgery were categorised in the postoperative radiotherapy (PORT) group.

Patterns of use of radiotherapy in rectal cancer were then investigated in relation to both the characteristics of the patients, their tumours, the interval to surgery and their management. The statistical significance of any differences in the type of radiotherapy used was assessed using the chi-squared test.

The work was given ethical approval by the East of Scotland Research Ethics Service (LR/08/S0501/66).

## Results

In total, 9201 individuals were identified within the NCDR as having undergone major resection for a first primary rectal cancer diagnosed in the study period. Overall, 4616 (50.2%) of this population did not receive any radiotherapy in the management of their primary disease. By contrast, 1113 (12.1%) received SCRT-I, 110 (1.2%) SCRT-D, 2713 (29.5%) LCCRT, 435 (4.7%) ORT and 214 (2.3%) PORT.

The characteristics of the population in relation to their treatment group are shown in Table 1. The use of radiotherapy decreased with age, with 60.5% of those less than 60 years of age at diagnosis receiving some form of radiotherapy compared with 28.1% of those aged over 80 years. Radiotherapy was also used more frequently in men than women (51.8% versus 46.1%). The use of radiotherapy increased with increasing stage (35.3% in Dukes A versus 54.5% in Dukes C) and also increased in relation to increasing socioeconomic deprivation, with 46.8% of those residing in the most affluent areas receiving the treatment compared with 55.0% in the least affluent areas. By contrast, rates of use of radiotherapy decreased in relation to increasing comorbidity, being used in 51.9% of those with a Charlson score of 0 compared with 28.7% of those with a score of 3 or more. Some form of radiotherapy was also used more frequently in those undergoing abdominoperineal resection compared with other types of major resection.

**Fig 1:**
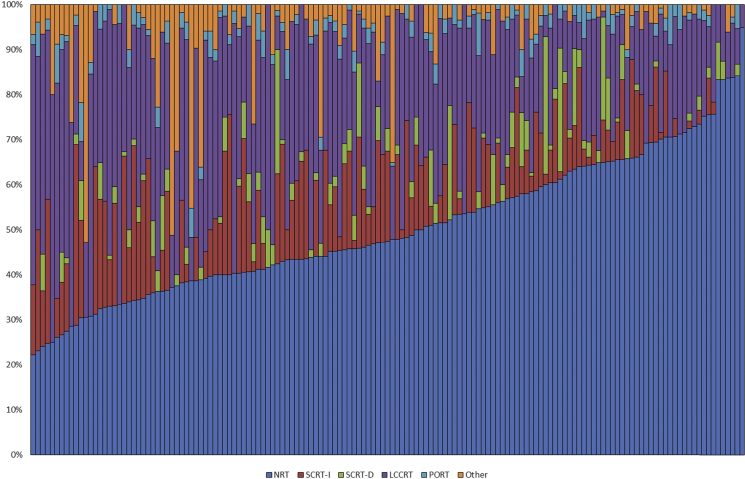
The proportion of individuals with surgically resected rectal cancer in each of the radiotherapy categories across all colorectal multidisciplinary teams in the English National Health Service.

Significant variation in the use of the different modalities of radiotherapy for the management of rectal cancer was seen across English NHS Trusts (Figure 1). The proportion of individuals in each Trust who did not receive any radiotherapy ranged between 22.2% and 94.9%. Equally, there were significant differences in the deployment of the different types of radiotherapy, with SCRT-I use ranging from 0.0% to 40.2%, SCRT-D from 0.0% to 10.0% and LCCRT from 5.1% to 62.5%. A few Trusts also seemed to apply a relatively high number of non-standard regimens, with the proportion of people in the ORT category ranging across Trusts from 0.0% to 52.8%. Twenty-three Trusts used a non-standard regimen in more than 10% of their cases. PORT was used infrequently in all Trusts, with a maximum use of 11.1%.

**Fig 2:**
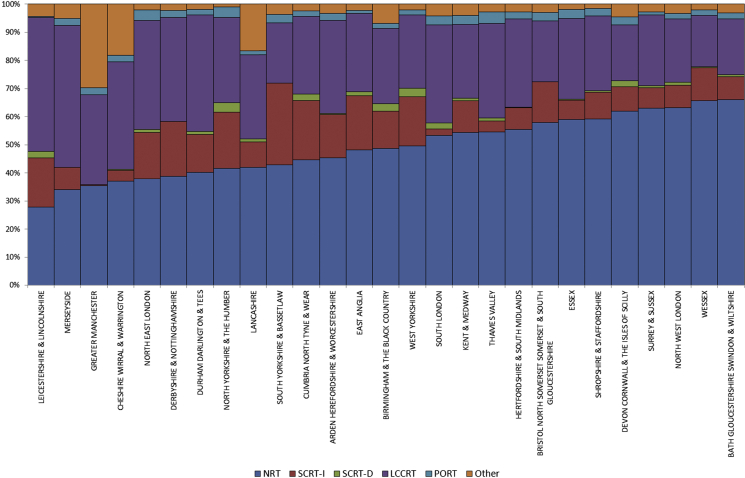
The proportion of individuals with surgically resected rectal cancer in each of the radiotherapy categories across all the Local Area Teams of the English National Health Service.

A significant, although slightly less marked variation was observed across the larger aggregation of MDTs within the English NHS's Local Area Teams (Figure 2). The proportion of individuals across the Local Area Teams who did not receive any radiotherapy ranged between 27.8% and 66.1%, but, as previously, there was also significant variation in the types of radiotherapy delivered. The proportion receiving SCRT-I ranged between 0.4% and 19.3%, SCRT-D between 0.0% and 3.4%, LCCRT between 18.1% and 50.5%, PORT between 0.4% and 4.1% and ORT between 1.1% and 29.6%.

**Fig 3:**
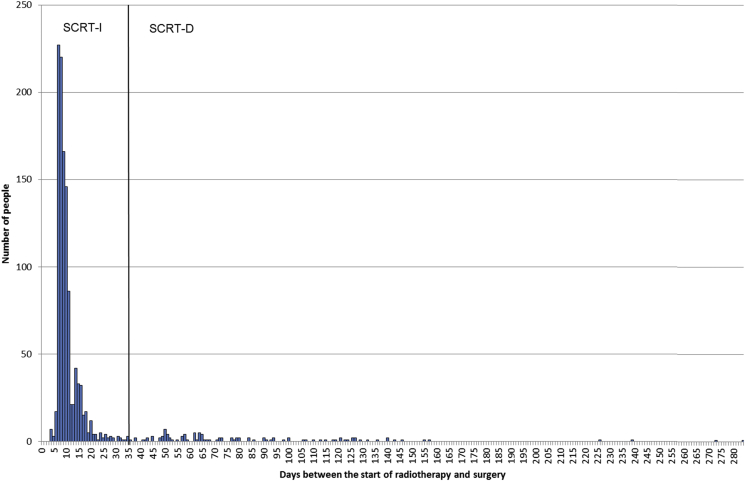
The number of individuals attending hospital for short-course radiotherapy in relation to the interval between the start of radiotherapy and surgery.

**Fig 4:**
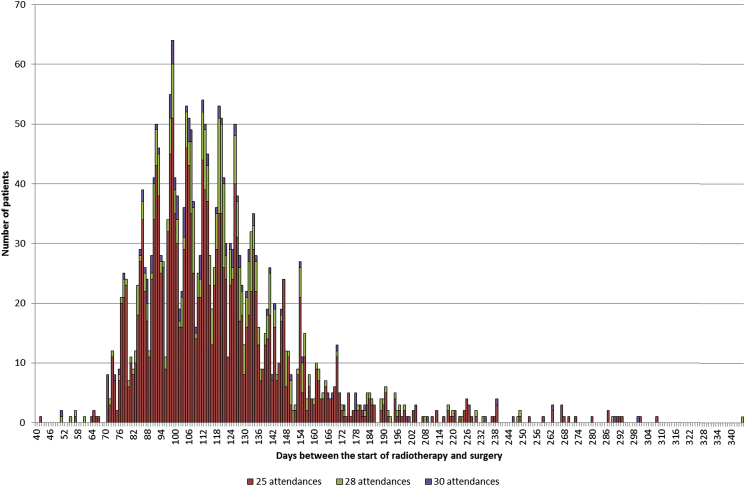
The number of individuals attending hospital for long-course chemoradiotherapy in relation to the interval between the start of radiotherapy and surgery and the total number of attendances.

**Fig 5:**
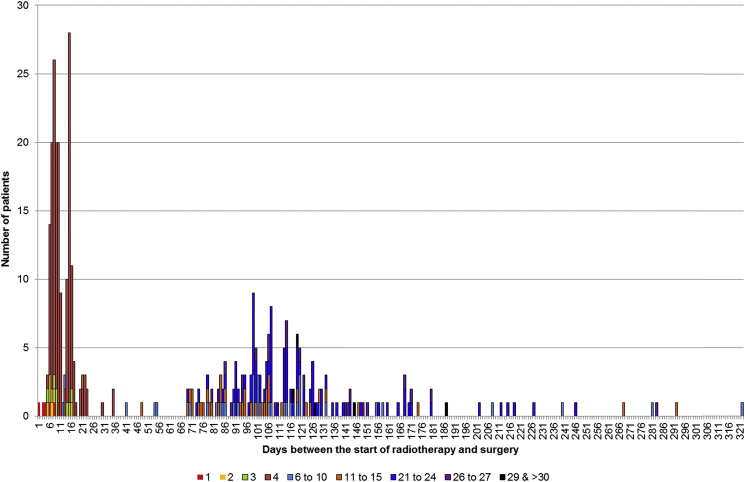
The number of individuals attending hospital for a non-standard radiotherapy regimen in relation to the interval between the start of radiotherapy and surgery and the number of attendances made.

There was also significant variation in practice in relation to the interval between the start of radiotherapy and surgery. Figure 3 shows the distribution in the number of days for this interval for individuals undergoing some form of SCRT. Most of the population who underwent SCRT-I surgery were resected within 14 days of the start of their radiotherapy, with a median interval of 9 days (interquartile range 8–11). There was, however, a considerable range in practice, with 113 people having 35 days or more between the start of radiotherapy and surgery (i.e. SCRT-D) and 34 people having an interval of 100 days or more. The median interval for this SCRT-D group was 72 days (interquartile range 52–118). The variation was also significant in the LCCRT group (Figure 4), with the interval between the start of radiotherapy and surgery ranging from 42 to 335 days. The median in the LCCRT category was 113 days (interquartile range 98–133). Figure 5 shows the distribution of the 435 people in the ORT category in relation to both the interval between radiotherapy and surgery and the total number of attendances to hospital. Most of the cases in this group arose from individuals attending hospital four or fewer times and receiving their subsequent surgery within 35 days of the first attendance. A further 253 individuals had intervals of greater than 35 days from the start of their radiotherapy to surgery and most of this group had greater than five attendances.

## Discussion

This retrospective population-based study is the first to provide a comprehensive national perspective on the use of radiotherapy in the management of surgically treated rectal cancer patients across England. Overall, 49.8% of the population received some form of radiotherapy, but there was variation across the population, with radiotherapy used more commonly in men and in those receiving abdominoperineal resection and less commonly in the elderly and those with comorbid disease. Significant variation in practice was also observed across the English NHS, irrespective of patient case mix, with regards to both the type of radiotherapy used and the interval between its initiation and surgery.

These data can be compared with those in the National Bowel Cancer Audit Project [29]. This voluntary audit captures data on around 86% of colorectal cancer patients treated in the UK, but its prime focus is surgery and only limited information is available within it on the use of radiotherapy. In the 2012 report (based on cases diagnosed in 2010/11), radiotherapy was used in a lower proportion of rectal cancer cases than in this study (41.7% versus 49.3%), with short course being used in 14.8% of cases, long course in 24.2% and postoperative in 1.7%. Radiotherapy was not reported or not given in 59.4% of cases. The data analysis cannot discriminate between these two responses. The audit indicated variation between Trusts (rates of use varied between 0 and 93%), but because of the voluntary nature of the audit and the lack of clarity of whether the treatment was not given or simply not reported it is hard to draw any firm conclusions. Significant variation in practice has also been observed across Welsh colorectal MDTs [30]. The current study provides, on a much larger sample, more robust population-based data for England and confirms significant variation in national patterns of practice.

This study not only showed variation in whether radiotherapy was used but also in what type was delivered and how long the interval was between the start of the radiation and surgery. To our knowledge no data have previously been published quantifying the extent of this variation across the English NHS. All these observed variations in radiotherapy usage were seen despite the routine weekly colorectal MDT meetings, which occur across the NHS, in which clinical and radiological staging investigations, including pelvic MRI, are reviewed to determine the selection of patients for preoperative treatment. MDTs are, therefore, adopting very different treatment strategies. How can this wide variation in radiotherapy usage be explained? A number of factors may have influenced MDT decisions. First, the Colorectal Improving Outcomes Guidance from 2004 [31] (which would have been relevant to the period covered by this study) recommended the use of either short-course preoperative radiotherapy or initial surgery with selective use of postoperative CRT based on involvement of the circumferential resection margin according to each MDT's defined unit policy. Only a minority of patients who undergo initial surgery will have an involved circumferential resection margin.

Second, there has been a growing body of evidence that preoperative radiotherapy lowers the risk of rectal cancer recurrence without any measurable impact on overall survival in moderate risk disease accompanied by lower rates of local recurrence with total mesorectal excision (TME) alone [2], [3], [7]. The quality of surgery in a unit was probably also relevant, with a correlation existing between lower local recurrence rates with better planes of surgical excision [9]. Finally there was also increasing concern that the addition of preoperative radiotherapy may lead to increased long-term toxicity [10], [11], [32], [33]. The weight each MDT placed on these different factors may account for the variability in practice observed.

Further evidence has been published relevant to the optimal use of radiotherapy in rectal cancer management subsequent to the study period. For example, several relevant phase III trials reported their outcomes after 2004 and new National Institute for Health and Care Excellence (NICE) colorectal cancer guidelines incorporating their findings were published in 2011 [34]. These guidelines define three risk groups for local recurrence after rectal cancer resection based on the pelvic MRI findings. Surgery alone is recommended for the low-risk group. SCRT or LCCRT should be considered for the medium-risk group and LCCRT for the high-risk group.

Several international studies have investigated the variation in use of radiotherapy for rectal cancer [16], [18], [19], [20], [35], [36], [37], [38]. Van Leersum *et al.* [38] reported a population-based study from the Netherlands between 2009 and 2011 and found that 85% of patients received preoperative radiotherapy. This is a significantly greater proportion than these English data indicate, but the Netherlands guidelines recommend the use of preoperative radiotherapy for all patients except those with T1N0 disease. By contrast, the recently published European Society of Medical Oncology rectal cancer radiotherapy guidelines [39] recommend a similar approach to those published by NICE in 2011 [34].

The significant variation in the range of intervals between the start of radiation and surgery is also of interest. The evidence based around what constitutes the optimal interval in either SCRT or LCCRT is relatively weak [39], [40], [41], [42] and it seems that, in the absence of definitive data, the practice patterns of MDT's are divergent. This may be further exemplified by the unusual spread of attendance patterns in the ORT group. A high proportion of this category attended hospital four times, suggesting the use of a four-fraction protocol. Although such a regimen is not widely used, the northwest rectal cancer randomised trial showed a significant reduction in local recurrence with 20 Gy in four fractions [43]. Alternatively, the unusual attendance patterns observed may indicate the use of different fractionation regimens or that patients discontinued treatment due to the toxicity of other medical events. These data cannot currently provide the detailed information to determine the cause or causes.

Indeed, a significant limitation of this study was the quality and extent of the data available in the RTDS. Numerous weaknesses were identified, including poor recording of the site of treatment, limited and unreliable information on treatment intent and, in certain centres, multiple episodes of care for a single course of treatment. However, linkage of the RTDS to other data sources available in the NCDR (notably cancer registry and HES data) alongside detailed clinical review and analysis did enable courses of radiotherapy to be related to both definitive diagnoses and surgical information. Patterns of care could then be quantified. A future development of the RTDS should, however, seek to both extend the data scope and its quality so that such robust analyses assessing the effect of variation in the time between radiotherapy and surgery initiation can be quantified and guidance produced to define optimal practice.

Recent changes in clinical practice may also alter future radiotherapy uptake [44]. This includes an increasing use of extra-levator abdominoperineal excision [45] with its reported reduced risks of resection margin involvement and specimen perforation, which may in turn reduce the use of preoperative radiotherapy. Conversely, the watch and wait option [46] using definitive CRT without surgery, attempting to delay or avoid major surgical resection for selected patients may be chosen by some MDTs [47]. This latter approach is not supported, however, by the 2011 NICE guidelines [34] and its evidence base remains inconclusive [48]. Analysis of further NCDR data to investigate how patterns of radiotherapy use change over time are, therefore, intended.

Although the present study has shown significant variation in the use of radiotherapy, it is unable to determine what influence this is having on locoregional failure or patient-reported outcomes. What is the correct balance between the benefits and risks of preoperative radiotherapy? Randomised trial evidence suggests that radiotherapy may reduce the risk of local recurrence, but it does not influence long-term survival. However, the addition of radiotherapy increases long-term side-effects [32], [33], [49], [50]. Without further information, quantifying both these positive and negative consequences of radiotherapy use, it is impossible to assess the effect of the variation in radiotherapy usage on patients or the NHS and this may increase rather than minimise variation in practice.

Although MDTs now routinely collect considerable information on the process of treatment, including histopathological assessment of the resected specimen, there is no systematic and prospective approach to record the timing and pattern of failure after rectal cancer resection and patient-reported outcomes. Extending the capture of such robust data to enable population-based assessment of the true effect of treatment variation on patient outcomes is vital if the NHS is to offer the best possible service. Our approach will be used to monitor the influence of the 2011 NICE guidelines. However, optimising the recommendations for the use of preoperative radiotherapy in future guidelines will depend on robust data linkage of radiotherapy data to both validated patient-reported outcomes and local recurrence rates. Additional linkage to fully completed Royal College of Radiologists and Pathologists minimum reporting datasets for each tumour would also give further insights to the choices made at MDT meetings.

## Conclusion

This population-based study has shown a wide variation in both the use of radiotherapy and radiotherapy schedules across the English NHS. Prospective population-based collection of locoregional recurrence, patient-reported toxicity and radiology and pathology datasets is required to understand and improve patient selection for preoperative radiotherapy, reduce variation in treatment and improve outcomes.

## Figures and Tables

**Table 1:** Characteristics of the study population

Characteristic		NRT		SCRT-I		SCRT-D		LCCRT		Other		PORT		Any radiotherapy		Total
*n*	%	*n*	%	*n*	%	*n*	%	*n*	%	*n*	%
Age group	≤60	929	39.6	282	12.0	15	0.6	951	40.5	109	4.6	63	2.7	1420	60.5	2348
61–70	1484	48.6	377	12.3	27	0.9	933	30.5	166	5.4	71	2.3	1574	51.5	3056
71–80	1509	53.3	362	12.8	40	1.4	731	25.8	129	4.6	61	2.2	1323	46.7	2832
>80	694	71.9	94	9.7	28	2.9	98	10.2	31	3.2	20	2.1	271	28.1	965
Gender	Male	2889	48.2	773	12.9	69	1.2	1849	30.8	276	4.6	138	2.3	3105	51.8	5994
Female	1727	53.9	340	10.6	41	1.3	864	26.9	159	5.0	76	2.4	1480	46.1	3207
Dukes stage at diagnosis	A	1289	64.7	253	12.7	13	0.7	345	17.3	76	3.8	17	0.9	704	35.3	1993
B	1239	54.7	282	12.5	31	1.4	559	24.7	107	4.7	46	2.0	1025	45.3	2264
C	1441	45.5	375	11.8	32	1.0	1057	33.4	164	5.2	97	3.1	1725	54.5	3166
D	299	50.1	39	6.5	9	1.5	191	32.0	34	5.7	25	4.2	298	49.9	597
Unknown	348	29.5	164	13.9	25	2.1	561	47.5	54	4.6	29	2.5	833	70.5	1181
Charlson comorbidity score	0	3645	48.1	957	12.6	91	1.2	2349	31.0	355	4.7	176	2.3	3928	51.9	7573
1	655	57.8	111	9.8	12	1.1	268	23.7	63	5.6	24	2.1	478	42.2	1133
2	204	60.4	32	9.5	5	1.5	72	21.3	14	4.1	11	3.3	134	39.6	338
≥3	112	71.3	13	8.3	2	1.3	24	15.3	3	1.9	3	1.9	45	28.7	157
IMD income category	Most affluent	1072	53.2	226	11.2	19	0.9	542	26.9	109	5.4	46	2.3	942	46.8	2014
2	1115	51.9	279	13.0	20	0.9	622	29.0	74	3.4	37	1.7	1032	48.1	2147
3	995	50.4	241	12.2	21	1.1	577	29.2	92	4.7	49	2.5	980	49.6	1975
4	816	48.2	212	12.5	28	1.7	521	30.8	70	4.1	46	2.7	877	51.8	1693
Least affluent	618	45.0	155	11.3	22	1.6	451	32.9	90	6.6	36	2.6	754	55.0	1372
Operation type	APE	550	25.4	334	15.4	45	2.1	1049	48.4	160	7.4	31	1.4	1619	74.6	2169
AR	3191	57.9	662	12.0	44	0.8	1312	23.8	214	3.9	90	1.6	2322	42.1	5513
Hartmanns	407	54.1	62	8.2	14	1.9	195	25.9	38	5.1	36	4.8	345	45.9	752
Other	468	61.0	55	7.2	7	0.9	157	20.5	23	3.0	57	7.4	299	39.0	767
Total		4616	50.2	1113	12.1	110	1.2	2713	29.5	435	4.7	214	2.3	4585	49.8	9201

NRT, no radiotherapy; SCRT-I, short-course radiotherapy with immediate surgery; SCRT-D, short-course radiotherapy with delayed surgery; LCCRT, long-course chemoradiotherapy; ORT, other radiotherapy; PORT, postoperative radiotherapy; IMD, index of multiple deprivation; APE, abdominoperineal excision; AR, anterior resection.
